# A Combination of CRISPR/Cas9 and Standardized RNAi as a Versatile Platform for the Characterization of Gene Function

**DOI:** 10.1534/g3.116.028571

**Published:** 2016-06-07

**Authors:** Sebastian Wissel, Anja Kieser, Tetsuo Yasugi, Peter Duchek, Elisabeth Roitinger, Joseph Gokcezade, Victoria Steinmann, Ulrike Gaul, Karl Mechtler, Klaus Förstemann, Jürgen A. Knoblich, Ralph A. Neumüller

**Affiliations:** * Institute of Molecular Biotechnology Austria, 1030 Vienna, Austria; † Gene Center, Ludwig-Maximilians-University Munich, 81377, Germany; ‡ Institute of Molecular Pathology, 1030 Vienna, Austria

**Keywords:** CRISPR, stem cell, *Drosophila*, loss-of-function

## Abstract

Traditional loss-of-function studies in *Drosophila* suffer from a number of shortcomings, including off-target effects in the case of RNA interference (RNAi) or the stochastic nature of mosaic clonal analysis. Here, we describe minimal *in vivo* GFP interference (miGFPi) as a versatile strategy to characterize gene function and to conduct highly stringent, cell type-specific loss-of-function experiments in *Drosophila*. miGFPi combines CRISPR/Cas9-mediated tagging of genes at their endogenous locus with an immunotag and an exogenous 21 nucleotide RNAi effector sequence with the use of a single reagent, highly validated RNAi line targeting this sequence. We demonstrate the utility and time effectiveness of this method by characterizing the function of the Polymerase I (Pol I)-associated transcription factor *Tif-1a*, and the previously uncharacterized gene *MESR4*, in the *Drosophila* female germline stem cell lineage. In addition, we show that miGFPi serves as a powerful technique to functionally characterize individual isoforms of a gene. We exemplify this aspect of miGFPi by studying isoform-specific loss-of-function phenotypes of the *longitudinals lacking (lola)* gene in neural stem cells. Altogether, the miGFPi strategy constitutes a generalized loss-of-function approach that is amenable to the study of the function of all genes in the genome in a stringent and highly time effective manner.

*Drosophila* geneticists have developed a remarkable number of strategies to perform loss-of-function analyses and to characterize gene function (Bellen 2014). Most notably, these methods consist of mutant analysis (zygotic or clonal), transgenic RNAi, and related approaches (Venken and Bellen 2005; Caussinus *et al.* 2012; Mohr *et al.* 2014). The choice of the appropriate experimental strategy is often influenced by the availability of reagents. For example, the unavailability of a characterized mutant might prompt researchers to generate multiple independent transgenic RNAi lines. Conversely, the availability of a lethal mutant might entail mosaic clonal analyses in a developmental stage after the lethality phase (Lee and Luo 2001).

Importantly, these methods suffer from a number of shortcomings. The analysis of zygotic mutants is only possible for amorphic or hypomorphic mutations that permit mutant analysis at the developmental time point of interest. In addition, a complex combination of cell autonomous and nonautonomous effects might contribute to a phenotype in a zygotic mutant, obscuring the interpretation of the observed defects. Moreover, the genetic background of long-kept mutants can represent complex genetic interactions. This phenomenon was recently exemplified by the high frequency of mutations in the *lethal giant larvae* gene in *Drosophila* second chromosome stocks (Roegiers *et al.* 2009). Similarly, mosaic clonal analysis, despite being highly informative in many experimental settings, suffers from the stochastic nature of mitotic recombination. This entails tedious work to identify a high enough number of clones in the desired cell type in order to be able to reach a statistically sound conclusion. In addition, phenotypes affecting the entire organ or a large cell population can only be studied in a limited set of tissues (*e.g.*, the *Drosophila* eye). Large numbers of clones can be generated using the FRT-FLP system (Newsome *et al.* 2000), but this system too relies on random clone induction, which can result in a variable frequency of homozygous mutant cells in the domain under which the Flipase is expressed.

RNAi-mediated knockdown constitutes an alternative loss-of-function strategy. In *Drosophila*, transgenic RNAi permits the knockdown of genes in a cell type-specific manner mediated by the UAS-Gal4 system (Brand and Perrimon 1993). Serious concerns about off-target effects have cast doubt over the specificity of this method [reviewed in Mohr *et al.* (2014)]. A recent study by Bellen and coworkers systematically compared RNAi and mutant phenotypes for genes on the *Drosophila* X chromosome and found that chemical mutagenesis and RNAi screens discover largely distinct sets of genes (Yamamoto *et al.* 2014). This and other data strongly argue that large-scale RNAi screens contain a high number of false positive or false negative hits. Thus, RNAi phenotypes need to be validated (for example by coexpression of an RNAi insensitive version of the gene) if used on a single gene level. Consequently, the faithful interpretation of RNAi data from large-scale approaches is only possible in a more aggregated form (for example at the level of protein complexes) (Mohr *et al.* 2014). We and others have recently developed a standardized RNAi based strategy, termed *in vivo* GFP interference (iGFPi) (or tag-mediated loss-of-function) (Pastor-Pareja and Xu 2011; Neumuller *et al.* 2012) to study loss-of-function phenotypes in a highly stringent manner. This method is based on the concept of using a sequence exogenous to the model species under investigation to knockdown genes of interest with RNAi constructs that by themselves do not induce phenotypes and can thus be considered off-target free. This methodology has been used for eGFP protein trap lines that were generated by random insertions of synthetic exons consisting of splice donor-eGFP-splice acceptor sequences into genes (Kelso *et al.* 2004; Buszczak *et al.* 2007; Quinones-Coello *et al.* 2007). The splicing of eGFP into the endogenous mRNA of a gene thus serves as an exogenous RNAi target sequence. We have generated a set of microRNA-based anti-eGFP shRNAs which permit, by using the UAS-Gal4 system (Brand and Perrimon 1993), cell type-specific loss-of-function of all the existing eGFP protein trap lines with a single standardized RNAi reagent (Neumuller *et al.* 2012).

The major drawbacks of this method are: i) the low number of eGFP tagged protein trap lines that exist in *Drosophila* and ii) the *a priori* inaccessibility of intronless genes (or genes with small introns) to protein tagging by random insertion of synthetic exons. Although recent efforts have extended the number of endogenously tagged proteins (Venken *et al.* 2011; Lowe *et al.* 2014; Lye *et al.* 2014; Dunst *et al.* 2015; Nagarkar-Jaiswal *et al.* 2015), the lack of readily available resources for protein trapped flies has hindered this method from becoming a generalized strategy for loss-of-function studies.

*Drosophila* germline and neural stem cells (GSCs and NSCs) (Figure 1A) are excellent model systems to study stem cell self-renewal in a genetically highly accessible species. GSCs are attached to a stemness-promoting niche and divide asymmetrically to generate another GSC and a differentiating cystoblast. The cystoblast divides four times with incomplete cytokinesis to form 4-cell, 8-cell, and 16-cell cystocytes. Among these 16 cells, one cell is destined to become the future oocyte whereas the remaining cells will endoreplicate to form polyploid support cells. Likewise, NSCs divide asymmetrically to generate another stem cell and a transit-amplifying cell termed the ganglion mother cell (GMC) in type I, and intermediate neural progenitor cell (INP) in type II, neuroblast (Nb) lineages. GMCs and INPs have limited replicative potential and ultimately generate neurons. In both lineages, stem cells possess a higher cellular growth rate than their immediate daughter cells. The maintenance of this high growth rate is required for stem cell self-renewal. GSCs and NSCs asymmetrically retain nucleolar proteins upon division (Fichelson *et al.* 2009; Zhang *et al.* 2014) to ensure a high growth rate. Moreover, cell growth inhibitory factors, including Mei-P26 and Brain tumor (Brat), accumulate in cystocytes and GMCs/INPs relative to the respective stem cells. Consistently, GSCs and NSCs possess bigger nucleoli, the centers of Pol I transcription and ribosome biogenesis, compared to their immediate progeny cells (Neumuller *et al.* 2008). We have previously published systematic analyses of GSC and NSC self-renewal using genome-scale transgenic RNAi. Only few of these reported stem cell factors have been rigorously validated owing to the lack of suitable mutants or independent RNAi reagents.

**Figure 1:**
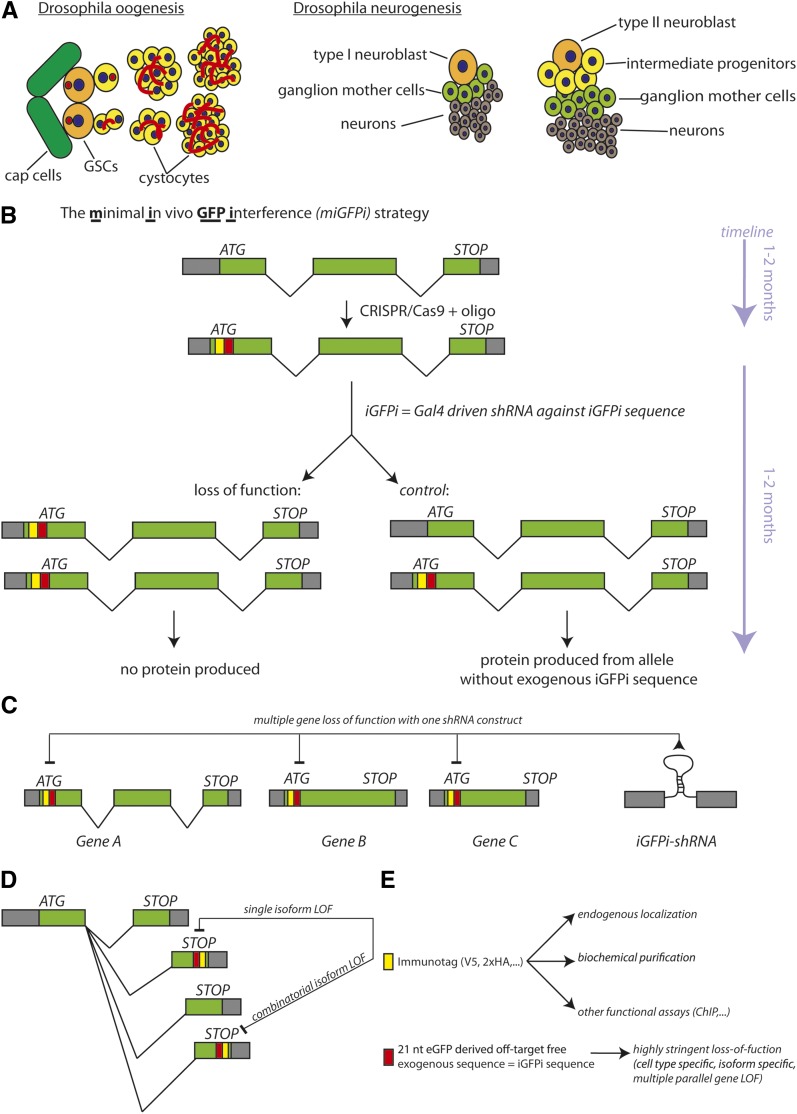
miGFPi as a versatile strategy to characterize gene function. (A) Schematic drawing of GSC and NSC lineages. GSCs and NSCs divide asymmetrically to generate another stem cell and a differentiating daughter cell. Differentiating daughter cells have limited proliferative potential. GSC differentiating daughter cells divide four times with incomplete cytokinesis to generate a 16-cell cyst. NSCs generate ganglion mother cells (GMCs) or intermediate neural progenitor cells (INPs) that generate postmitotic neurons after a limited number of divisions. (B) Outline of the miGFPi strategy. CRISPR/Cas9-mediated insertion of an oligo into the coding sequence of a gene. The oligo contains one component encoding for an immunotag (e.g.: V5 or HA) and one component constituting an RNAi target sequence derived from eGFP. When homozygous, this chromosome allows for RNAi-mediated loss-of-function of the gene. When heterozygous, the nontagged allele of the gene is unaffected by RNAi and preserves gene function, serving as a control. (C) miGFPi allows for simultaneous loss of function of numerous genes into which the oligo has been inserted. (D) miGFPi allows for loss-of-function studies of individual or combinations of isoforms from a gene. (E) Potential applications of the two components of the oligo-based tag. CRISPR, clustered regularly interspaced short palindromic repeats; eGFP, enhanced GFP; GFP, green fluorescent protein; GSC, germline stem cell; iGFPi, *in vivo* GFP interference; LOF, loss-of-function; miGFPi, minimal *in vivo* GFP interference; NSC, neural stem cell; RNAi, RNA interference; shRNA, short hairpin RNA.

Here, we report the “minimal *in vivo* GFP interference” (miGFPi) method, which constitutes an advancement of iGFPi into a generalized strategy amenable, in principle, to all genes. The new method combines highly efficient CRISPR/Cas9-based integration of a 21 nucleotide stretch, which serves as an exogenous RNAi effector sequence into the endogenous locus of a gene of interest with the use of a highly validated RNAi line against that sequence. Along with the 21 nucleotide effector sequence, we integrate an immunotag (*e.g.*, V5 or HA tag) into the coding sequence of a gene of interest. With this approach, it is possible to tag multiple genes or multiple gene isoforms with different immunotags but the same RNAi effector sequence. This allows the induction of simultaneous loss-of-function of the tagged genes or gene isoforms, while the individual immunotag can be used for localization as well as biochemical studies. This improved method allows for the generation of the relevant fly stocks for loss-of-function and functional analyses of individual genes in an 8-week period and, thus, serves as a generalized strategy for the characterization of gene function in a time efficient and highly stringent manner.

## Materials and Methods

### CRISPR/Cas9 tagging

All loci for the genes modified by the CRISPR/Cas9 mentioned in this study were sequenced at the gRNA region to ensure matching sequences between gRNA and genomic locus. gRNA constructs were cloned into the pDCC6 plasmid (Gokcezade *et al.* 2014) and injected into embryos of an isogenized *w^1118^* fly strain. Fly crosses for CRISPR/Cas9 tagging were conducted as previously indicated (Gokcezade *et al.* 2014). Successfully targeted flies were identified by PCR screening, with one primer binding upstream of the gRNA region and one primer binding in the insert. Homozygous viable targeted fly strains were used to amplify the modified region by PCR followed by subsequent sequencing. Sequences of all oligos and primers used can be found in Supplemental Material, Table S1 and File S1. It is worth noting that miGFPi might also work by directing RNAi against the V5 or HA sequence. We opted for an eGFP-based effector sequence in this study because of the availability of our previously validated eGFP shRNA lines in NSCs and GSCs (Neumuller *et al.* 2012). A bioinformatics analysis for different V5 sequences revealed sequences with and without predicted off-targets in the *Drosophila* genome. The top scoring RNAi compatible V5 sequences are listed in Table S1. Endogenously GFP tagged MESR4 cell lines were established following a procedure described in (Kunzelmann *et al.* 2016).

### Fly crosses and mutant analysis

All fly crosses were set up at 25° on standard fly food. For the analysis of germline phenotypes, F1 flies were collected after hatching and incubated at 29° for 4 d. Ovaries were dissected in PBS, fixed in 4% PFA in 0.1% Triton X-100 in PBS, and incubated with primary and secondary antibodies as previously described (Yan *et al.* 2014). For NSC analysis, crosses were set up at 25° and larvae were raised at 29°. Brains of wandering third instar larvae were dissected and further processed for immunofluorescence. Since wor-Gal4-mediated miGFPi depletion of *lola*-all was early lethal, the line was combined with a tubGal80^ts^ transgene. Crosses were set up at 18° and transferred to 29° after 4 d. Samples were mounted in Vectashield (containing DAPI); images were recorded on a Zeiss-LSM710 or LSM780 microscope and quantifications were made using Zeiss ZEN software.

### Fly strains used

The following fly strains were used: Valium20 eGFP-shRNA (Bloomington Stock Center number: 41556) (Neumuller *et al.* 2012); Nanos-gal4 VP16; w^1118^; worniu (wor)-Gal4; and wor-Gal4,ase-Gal80 (Neumuller *et al.* 2011). *MESR4* RNAi lines GL00462 and HMC04881 were kindly provided by the TRiP (Transgenic RNAi project, Harvard Medical School) (Perkins *et al.* 2015). Generation of *lola*-specific shRNA transgenic flies: synthesized oligos (see Table S1 for sequences) were annealed and cloned into the Walium20 vector according to protocols of The Transgenic RNAi project (http://www.flyrnai.org/).

### Cloning of MESR4 and Tif-1a

*MESR4* was assembled from a partial EST (LD04271) and a *de novo* synthesized fragment (see Table S1 for sequence information) for the C-terminal part of *MESR4* using Gibson assembly and cloned into an attP-containing pUASp vector. *Tif-1a* was PCR amplified from a cDNA library and cloned into the attP-containing pUASp vector. The S610A and S610D point mutations were generated by PCR amplification of the *Tif-1a* WT open reading frames with primers carrying the respective mutations. Transgenic flies were established using the attP2 (Markstein *et al.* 2008) landing site using standard procedures. Among different landing sites for *pUASp-Tif-1a* we opted for the attP2 (3rd chromosome) site, as a previously published *Tif-1a* overexpression line (2nd chromosome) generated by Zhang and coworkers was not able to rescue the *Tif-1a* miGFPi phenotype, presumably due to insufficient expression levels Zhang *et al.* 2014.

### Antibodies used

Mouse anti-V5 (1:400, Sigma-Aldrich V8012), mouse anti-1B1 (1:1, Developmental Studies Hybridoma Bank), goat anti-Vasa, mouse anti-Orb (1:20, Developmental Studies Hybridoma Bank), rabbit anti-Miranda (1:100), guinea pig anti-Miranda (1:200), rabbit anti-n-terminal lola (Giniger *et al.* 1994), mouse anti-HA (1:50), and rat anti-ELAV (1:50, Developmental Studies Hybridoma Bank).

### Quantitative real-time PCR

cDNA was generated using the iScript cDNA synthesis kit (BioRad) on TRIzol-extracted total RNA. qPCRs were done using iQ SYBR Green Supermix (BioRad) on a BioRad CFX96 cycler. Expression of each gene was normalized to Act5C, and relative levels were calculated using the ${\text{2}}^{\mathrm{-\Delta \Delta}{\text{C}}_{\text{T}}}$ method (Livak and Schmittgen 2001).

### Mass-spectrometry analysis

Plasmids encoding *actin 5c* promoter-driven *Gal4* and UAS-n-terminally GFP tagged *Tif-1a* were transfected into *Drosophila* S2 cells and precipitated using a GFP-specific nanobody (Chromotek GFP-Trap) coupled to agarose beads, using the manufacturers’ suggestions. The proteins were eluted from the beads by addition of 3 × 40 µl of 0.1 M Glycine pH 2. The eluate was neutralized by addition of 30 µl of 1 M Tris-Cl pH 8. Proteins were reduced with 0.2 mM Dithiothreitol (DTT, Roche Diagnostics) at 56° for 30 min and subsequently alkylated with 1 mM S-methyl methanethiosulfonate (MMTS; Fluka) in the dark at room temperature (RT) for 30 min. Proteins were digested with 400 ng of trypsin (Trypsin gold; Promega) at 37°. The digestion was stopped after 16 hr by adding 10 µl of 10% trifluoroacetic acid (TFA; Thermo Scientific).

The C-terminal tryptic peptide of *Tif-1a* (QFHFGSSP) was synthesized in its unmodified and phosphorylated (on S7) form using solid-phase Fmoc chemistry, purified using preparative reversed-phase chromatography, lyophilized, and subsequently characterized by MALDI-TOF- MS (using an ABI 4800 MALDI-TOF/TOF, SCIEX).

The digested protein samples and, subsequently, the synthetic peptides were analyzed by LC-MS/MS applying the same methods. The nano HPLC system used was an UltiMate 3000 HPLC RSLC nano system (Thermo Scientific) coupled to a Q Exactive Plus mass spectrometer (Thermo Scientific), equipped with a Proxeon nanospray source (Thermo Scientific). Peptides were loaded onto a trap column (Thermo Scientific, PepMap C18, 5 mm × 300 μm ID, 5 μm particles, 100 Å pore size) at a flow rate of 25 μl min^-1^ using 0.1% TFA as mobile phase. After 10 min, the trap column was switched in line with the analytical column (Thermo Scientific, PepMap C18, 500 mm × 75 μm ID, 3 μm, 100 Å). Peptides were eluted using a flow rate of 230 nl min^-1^ and a binary 180 min gradient. The gradient started with the mobile phases: 98% A (water/formic acid, 99.9/0.1, v/v) and 2% B (water/acetonitrile/formic acid, 19.92/80/0.08, v/v/v) increased to 35% B over the next 180 min, followed by a gradient in 5 min to 90% B, stayed there for 5 min, and decreased in 5 min back to the gradient 98% A and 2% B for equilibration at 30°.

The Q Exactive mass spectrometer was operated in data-dependent mode, using a full scan (m/z range 380–1650, nominal resolution of 70,000, target value 3E6) followed by MS/MS scans of the 12 most abundant ions. The default charge state was set to 3 and singly charged ions were excluded from fragmentation. MS/MS spectra were acquired using normalized collision energy 27%, isolation width of 2, and the target value was set to 1E5. Precursor ions selected for fragmentation (charge state 2 and higher) were put on a dynamic exclusion list for 30 sec. The underfill ratio was set to 20% resulting in an intensity threshold of 4e4. The peptide match feature and the exclude isotopes feature were enabled.

RAW-files were loaded into Proteome Discoverer (version 1.4.0.288, Thermo Scientific) and the MS/MS spectra were searched using MS Amanda (Version 1.4.14.3917) against Flybase (release FB2013_04, dmel_all-translation-r5.52) supplemented with common contaminants. The following search parameters were used: β-methylthiolation on cysteine was set as a fixed modification, oxidation on methionine, acetylation on lysine, phosphorylation on serine, and threonine and tyrosine were set as variable modifications. Monoisotopic masses were searched within unrestricted protein masses for tryptic peptides. The peptide mass tolerance was set to ± 8 ppm (parts per million) and the fragment mass tolerance to ± 20 ppm. The maximal number of missed cleavages was set to 2. The result was validated using the Percolator algorithm integrated in Proteome Discoverer. The localization of the sites of variable modifications within the peptides was performed with the tool ptmRS, integrated in Proteome Discoverer and based on phosphoRS.

The MS/MS spectra of the C-terminal peptide, both in the unmodified and phosphorylated form, were identified with weak MS Amanda scores, corresponding to a false discovery rate of higher than 5%. Therefore, the same tryptic peptide was chemically synthesized in its unmodified and phosphorylated form and analyzed with a similar LC-MS/MS method after the actual samples. Both the unmodified and modified synthetic peptides showed a similar elution time (data not shown) and highly similar MS/MS spectra as the corresponding endogenous *Tif-1a* peptides (shown for the phosphorylated peptides in Figure S1), which is a highly reliable confirmation of the correct identification of the peptide sequence.

### Data availability

The authors state that all data necessary for confirming the conclusions presented in the article are represented fully within the article.

## Results

### miGFPi demonstrates a role of Tif-1a in GSC maintenance

To overcome the intrinsic limitations of current loss-of-function approaches (including iGFPi) in *Drosophila*, we designed a generalized strategy that represents an “all-in-one platform” to characterize gene function (Figure 1, B–E). To allow for maximal flexibility and time efficiency, we chose a CRISPR/Cas9-oligo-based strategy for the insertion of a 66 nucleotide stretch into the coding region of a gene of interest. The 66 nucleotides encode for a V5 immunotag, which is followed by a 21 nucleotide sequence derived from eGFP (see Table S1 for details). Due to the availability of highly specific antibodies against the V5 immunotag, the resulting endogenously tagged protein can be used for studying protein expression as well as for biochemical assays. In addition, the availability of a highly specific and validated RNAi line for usage in GSCs and NSCs against the 21 nucleotide stretch enables off-target free knockdown of the endogenous mRNA. Importantly, this strategy combines several important improvements: i) the versatile CRISPR/Cas9-oligo-based insertion provides the desired flexibility that allows for the integration of the insert into intron-containing and intronless genes; ii) the precision of the oligo insertion allows for isoform-specific miGFPi based loss-of-function (Figure 1D); iii) the parallel integration of the same exogenous sequence into different genes or isoforms permits combined loss-of-function with one single shRNA reagent (Figure 1, C and D); and iv) the relevant fly stocks can be generated within 2 months and the method can therefore be used for medium throughput approaches.

**Figure 2:**
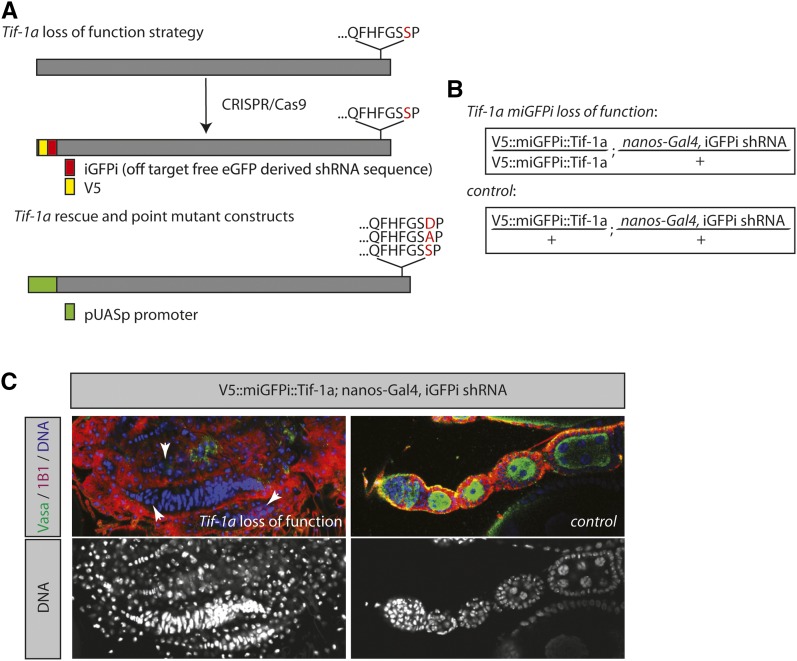
miGFPi unravels a requirement for *Tif-1a* in GSC maintenance. (A) miGFPi strategy for *Tif-1a* loss-of-function and rescue experiments (*Tif-1a* wild-type, S610A, and S610D sequences are shown at the C-terminus). (B) Relevant miGFPi genotypes for loss-of-function experiments. (C) *Tif-1a* is required for germline stem cell maintenance. Compared to the wild type (right), miGFPi-mediated depletion of *Tif-1a* results in a loss of all germline cells and empty ovarioles (white arrowheads). Blue (gray lower panels), DNA; Red, 1B1 [fusome and spectrosome marker (undifferentiated cells)], Green: Vasa (germline cell marker). CRISPR, clustered regularly interspaced short palindromic repeats; eGFP, enhanced green fluorescent protein; GSC, germline stem cell; iGFPi, *in vivo* GFP interference; miGFPi, minimal *in vivo* GFP interference; shRNA, short hairpin RNA.

To ask if this strategy works in principle, we turned to the gene *Tif-1a*. *Tif-1a* is a Polymerase I (Pol I)-specific transcription factor that is required for rDNA transcription. A high level of Pol I transcription and an associated high cell growth rate is required for the maintenance of GSCs (Neumuller *et al.* 2008, 2013; Fichelson *et al.* 2009; Zhang *et al.* 2014; Yan *et al.* 2014). In accordance with the notion that growth promoting factors are needed in the GSCs, a GSC loss phenotype for *Tif-1a* RNAi has recently been reported in a genome-scale RNAi screen for GSC self-renewal (Yan *et al.* 2014). To validate this phenotype and to test the miGFPi approach, we introduced 66 nucleotides encoding for a V5 immunotag and the eGFP-derived RNAi effector sequence (Figure 2A, see Table S1 for primer details) downstream of the START codon of *Tif-1a* using a recently published CRISPR/Cas9 system (Gokcezade *et al.* 2014). The resulting flies are homozygous, viable, and fertile.

To ask if the 21 nucleotide eGFP-derived sequence would enable highly specific phenotypic analyses, we conducted a series of loss-of-function and rescue experiments that underline the flexibility and stringency of miGFPi (Figure 2B) as described below. In humans, Tif-1a is a phosphoprotein. Serine 649 is phosphorylated in a cell growth-dependent manner by ERK (Zhao *et al.* 2003) and the phosphomutant form, Tif-1a S649A, is insufficient to stimulate Pol I transcription. We envisioned that the rescue of *Tif-1a* miGFPi, which resulted in a complete depletion of GSCs and consequently all germline cells (Figure 2C), with wild type and inactive *Tif-1a*, would be a good test for the specificity of the method. Evidence for the phosphorylation of Serine S610 of *Drosophila Tif-1a* exists in the literature [PhosphoPep database, Bodenmiller *et al.* (2007)]. Similar to the human system, the S610 site constitutes an ERK consensus site which is located at the very C-terminus of the protein.

**Figure 3:**
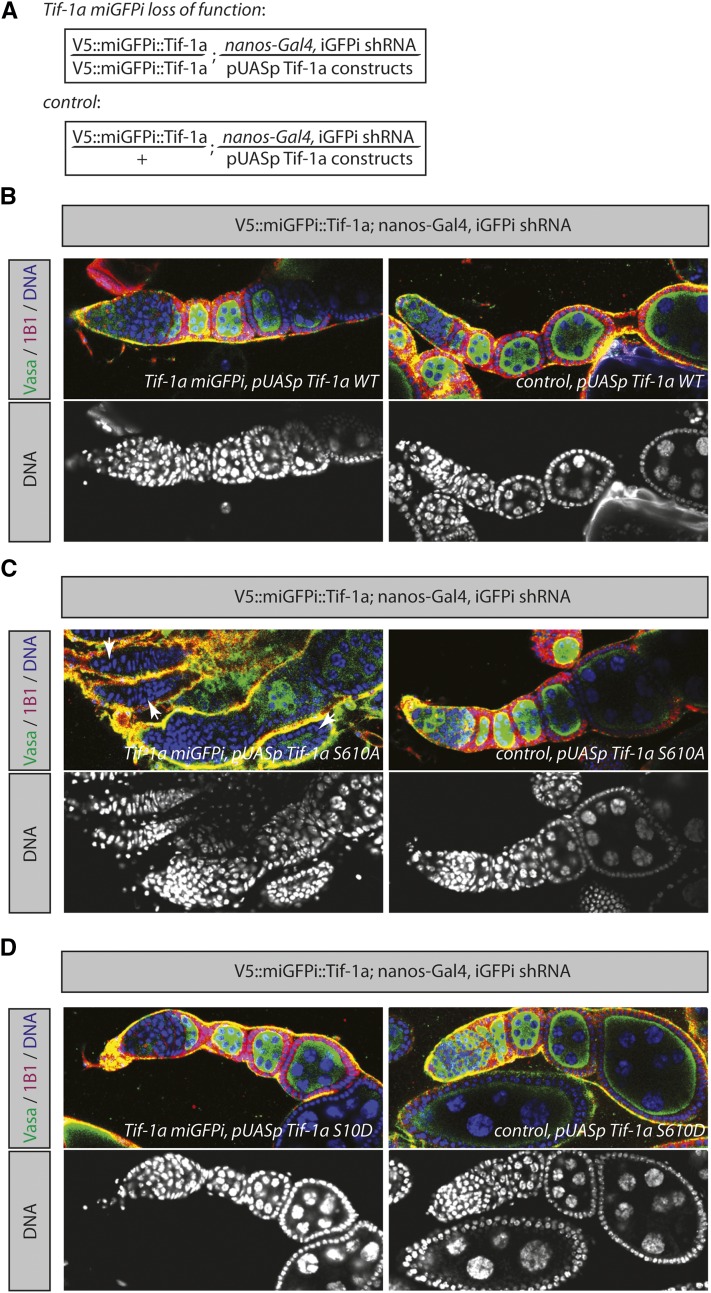
Rescue of the miGFPi-induced phenotypes. (A) Outline of the miGFPi genotypes for the *Tif-1a* rescue experiments. (B–D) Expression of a *Tif-1a* wild-type and *Tif-1a* S610D construct in the miGFPi *Tif-1a* background fully rescues the *Tif-1a* loss-of-function phenotype, whereas the miGFPi *Tif-1a* S610A ovaries contain empty ovarioles (white arrowheads) (for details see text). iGFPi, *in vivo* GFP interference; miGFPi, minimal *in vivo* GFP interference; shRNA, short hairpin RNA; WT, wild type.

To substantiate that S610 is phosphorylated, we precipitated GFP-tagged *Tif-1a* from *Drosophila* S2 cells and subjected it to mass spectrometry analysis. Consistent with the high throughput proteomics approach (Bodenmiller *et al.* 2007), we found peptides that demonstrate that *Drosophila* *Tif-1a* is phosphorylated at Serine 610 (Figure S1). We then constructed transgenic flies in which UAS-*Tif-1a* wild-type (WT), UAS-*Tif-1a* S610A (phosphomutant), and UAS-*Tif-1a* S610D (phosphomimetic) overexpression constructs are inserted at the exact same genomic location (Brand and Perrimon 1993; Markstein *et al.* 2008). miGFPi of *Tif-1a* resulted in a GSC loss phenotype, and consequently an ablation, of all germline cells (Figure 2C). Upon expression of *Tif-1a* WT in the *Tif-1a* miGFPi background, this phenotype is completely reversed and the ovaries are indistinguishable from the wild type (Figure 3, A and B). Conversely, upon expression of *Tif-1a* S610A in the miGFPi background, the overwhelming majority of ovarioles (70.6% empty, 48 out of 68 analyzed ovarioles) do not contain germline cells compared to control flies (0% empty, 0 out of 55 analyzed ovarioles), suggesting that the phosphomutant form cannot completely compensate for the loss of *Tif-1a* (Figure 3C). Similar to the WT situation, expression of S610D fully rescues the phenotype (Figure 3D). These data suggest that the miGFPi method is highly specific and reliable in studying loss-of-function phenotypes. In addition, the data suggest that miGFPi can be used to study the functional significance of individual amino acids and/or domains by expressing the appropriate constructs in a miGFPi background.

### miGFPi unravels a requirement for MESR4 in germline cell differentiation

**Figure 4:**
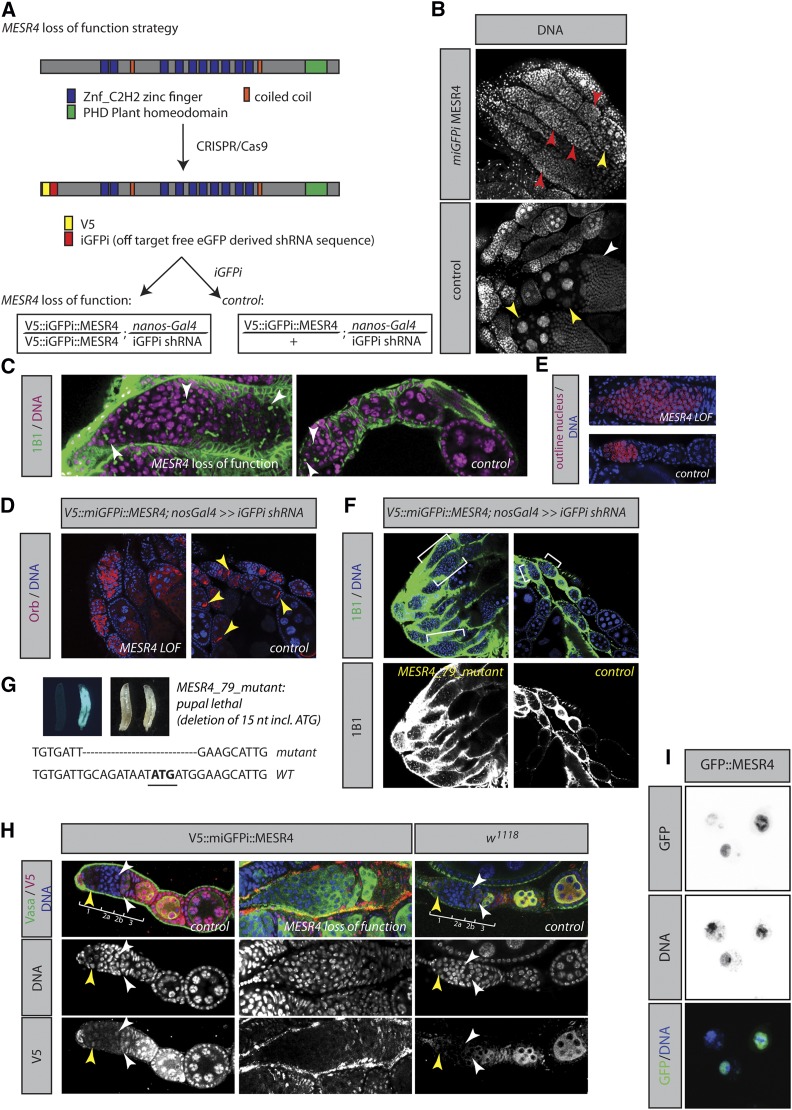
*MESR4* controls differentiation in the GSC lineage. (A) miGFPi strategy for *MESR4* loss-of-function. (B) Loss of *MESR4* function in the germline is associated with a decreased size of the ovaries and differentiation defects of germline cells [pseudo egg chambers containing undifferentiated cells (red arrowheads), polyploid nurse cells (yellow arrowheads), and maturing oocytes (white arrowhead)]. (C and E) The number of 1B1 positive cells in the germarium is expanded in *MESR4* miGFPi. (C) Green, 1B1; magenta, DNA. (E) Red: manual outline of nuclei of 1B1 positive cells of a *MESR4* miGFPi and wild-type germarium. (D) The oocyte-accumulating protein Orb does not enrich in specific cells in *MESR4* miGFPi as compared to the control (yellow arrowheads). (F) One copy of the *V5*::miGFPi::*MESR4* over *MESR4^79^* and nanos-Gal4-driven *iGFPi* shRNA results in the same phenotype as *MESR4* miGFPi. (G) Identification of a CRISPR/Cas9-generated *MESR4* mutant. *MESR4* mutants die at pupal stages. The genomic region flanking the gRNA was PCR amplified from homozygous *MESR4^79^* larvae and sequencing revealed a deletion of the start codon. The genotypes are: left larva, *MESR4^79^/MESR4^79^*; right larva, *MESR4^79^*/CyO- Kr-Gal4 UAS-GFP. (H) MESR4 is expressed in the germline. V5 staining of homozygous *V5*::miGFPi::*MESR4* reveals low expression of MESR4 in GSCs and early cystocytes. MESR4 is strongly induced in 16-cell cystocytes and remains highly expressed at later stages of differentiation yellow arrowheads: GSCs, white arrowheads: differentiating cells. (I) Endogenously, N-terminally GFP tagged MESR4 in *Drosophila* S2 cells localizes predominantly to the nucleus and is weakly detectable in the cytoplasm (blue, DNA; green, GFP::MESR4). CRISPR, clustered regularly interspaced short palindromic repeats; eGFP, enhanced GFP; GFP, green fluorescent protein; GSC, germline stem cell; iGFPi, *in vivo* GFP interference; miGFPi, minimal *in vivo* GFP interference; RNAi, RNA interference; shRNA, short hairpin RNA.

Because a requirement of Pol I transcription in GSC maintenance has been demonstrated before (Neumuller *et al.* 2008; Fichelson *et al.* 2009; Zhang *et al.* 2014; Yan *et al.* 2014), our *Tif-1a* experiments have “proof-of-concept” character. Therefore, we wanted to know if we could use the miGFPi strategy for loss-of-function studies of an as-yet uncharacterized gene in the GSC lineage. Out of a previously published RNAi screen in GSCs (Yan *et al.* 2014) we picked a putative transcriptional regulator, *misexpression suppressor of ras 4* (*MESR4*), for further analysis. The previously reported phenotype stems from the analysis of one RNAi line from the large-scale screen and is thus not validated (Yan *et al.* 2014). To test the validity of the initial observation that *MESR4* loss-of-function results in an expansion of undifferentiated cells in the GSC lineage, we employed miGFPi. Using CRISPR/Cas9, we integrated a V5 tag and the RNAi effector sequence after the START codon of *MESR4* (Gokcezade *et al.* 2014) (Figure 4A). The resulting flies are homozygous, viable, and fertile. We put the tagged *MESR4* into a genetic background in which the RNAi construct targeting the 21 exogenous nucleotides is expressed from the germline-specific *nanos-Gal4*. Depletion of *MESR4* resulted in sterile flies with ovaries dramatically reduced in size (Figure 4B). We did not detect mature eggs upon *MESR4* loss-of-function and found that 17.2% of the analyzed ovarioles (33 out of 192 ovarioles) contained pseudo egg chambers filled with 1B1 positive, fusome-containing undifferentiated cells (Figure 4, B and C). All analyzed *MESR4* loss-of-function ovarioles also contained pseudo egg chambers with polyploid nurse cells but absent oocytes. Consistent with the absence of mature eggs, the oocyte-specific marker Orb was mislocalized in 76% (57 out of 75) of the analyzed pseudo egg chambers in the *MESR4* miGFPi genotype as compared to the wild type (0 out of 18 analyzed egg chambers) (Figure 4D). These data suggest that *MESR4* is required for the differentiation of germline cells and either oocyte specification or oocyte survival.

In our miGFPi experiments, we further detected a highly penetrant phenotype in the germaria. The average length from *MESR4* miGFPi germaria was increased from 50.59 (± 2.272) μm in WT to 81.74 (± 11.516) μm (*n* > 10 germaria) (Figure 4E). Given the higher number of 1B1 positive cells in the germarium, we suspect that an expansion of cystocytes might be the underlying cause for this phenotype. These data are consistent with the observed phenotypes of two independent shRNA constructs targeting *MESR4* (Figure S2). In both cases, we detected an expansion of the number of undifferentiated, fusome-containing cystocytes in the germarium, and the co-occurrence of polyploid nurse cells and 1B1 positive cells containing pseudo egg chambers. We did not detect mature eggs in *MERS4* miGFPi or RNAi experiments, suggesting that *MESR4* is required for multiple processes in germline development, including oocyte specification and controlling the size of the transit amplifying cystocyte cell pool.

To gain further evidence for the specificity of the method, we combined miGFPi with mutant analysis. We generated a *MESR4* mutant using CRISPR/Cas9. The resulting flies (*MESR4^79^*) are pupal lethal and the zygotic mutants can thus not be used for phenotypic analysis in adult GSCs (Figure 4G). When *V5*::miGFPi::*MESR4*; nanos-Gal4, miGFPi-sh-RNA flies are crossed to *MESR4^79^* mutant flies, we observed the same ovarian phenotype as in the setting when *V5*::miGFPi::*MESR4* is homozygous (Figure 4F). In addition, we cloned the *MESR4* gene and expressed it in the *MESR4* miGFPi background to test the specificity of the method. Consistent with the previous results, we were able to fully rescue the *MESR4* miGFPi phenotype when we express wild-type *MESR4* in the miGFPi background. Altogether, these results suggest that the miGFPi strategy is specific and can elucidate the phenotype of previously uncharacterized genes in a highly stringent manner.

### MESR4 is a nuclear protein expressed in the germline

As our insert contains a V5 tag besides the RNAi target sequence, we wanted to ask if MESR4 is expressed in the germline as suggested by our loss-of-function analysis. V5 staining of homozygous *V5*::miGFPi::*MESR4* ovaries revealed that MESR4 is a predominantly nuclear protein with a small pool of protein detectable in the cytoplasm. In ovaries, MESR4 is expressed in both somatic and germline cells. MESR4 is weakly detectable in GSCs and their immediate daughter cells. In 16-cell cystocytes, MESR4 is strongly induced and remains highly expressed at all later stages of oogenesis (Figure 4H). Because all anti-V5 antibodies showed a weak cytoplasmatic background in the ovaries, we wanted to corroborate the finding that MESR4 is a predominantly nuclear protein. Therefore, we tagged *MESR4* at the endogenous locus in *Drosophila* S2 cells at the N-terminus (Kunzelmann *et al.* 2016). Consistent with the *in vivo* findings, GFP::MESR4 protein is detectable in the nucleus and a small pool of GFP::MESR4 localizes to the cytoplasm (Figure 4I). In summary, the MESR4 *in vivo* expression pattern is consistent with the observed miGFPi phenotype, and its subcellular localization is consistent with the proposed function of MESR4 as a chromatin regulator. Altogether, these experiments suggest that the composition of our exogenous sequence permits the rapid characterization of gene function.

### miGFPi permits efficient double mutant analysis

**Figure 5:**
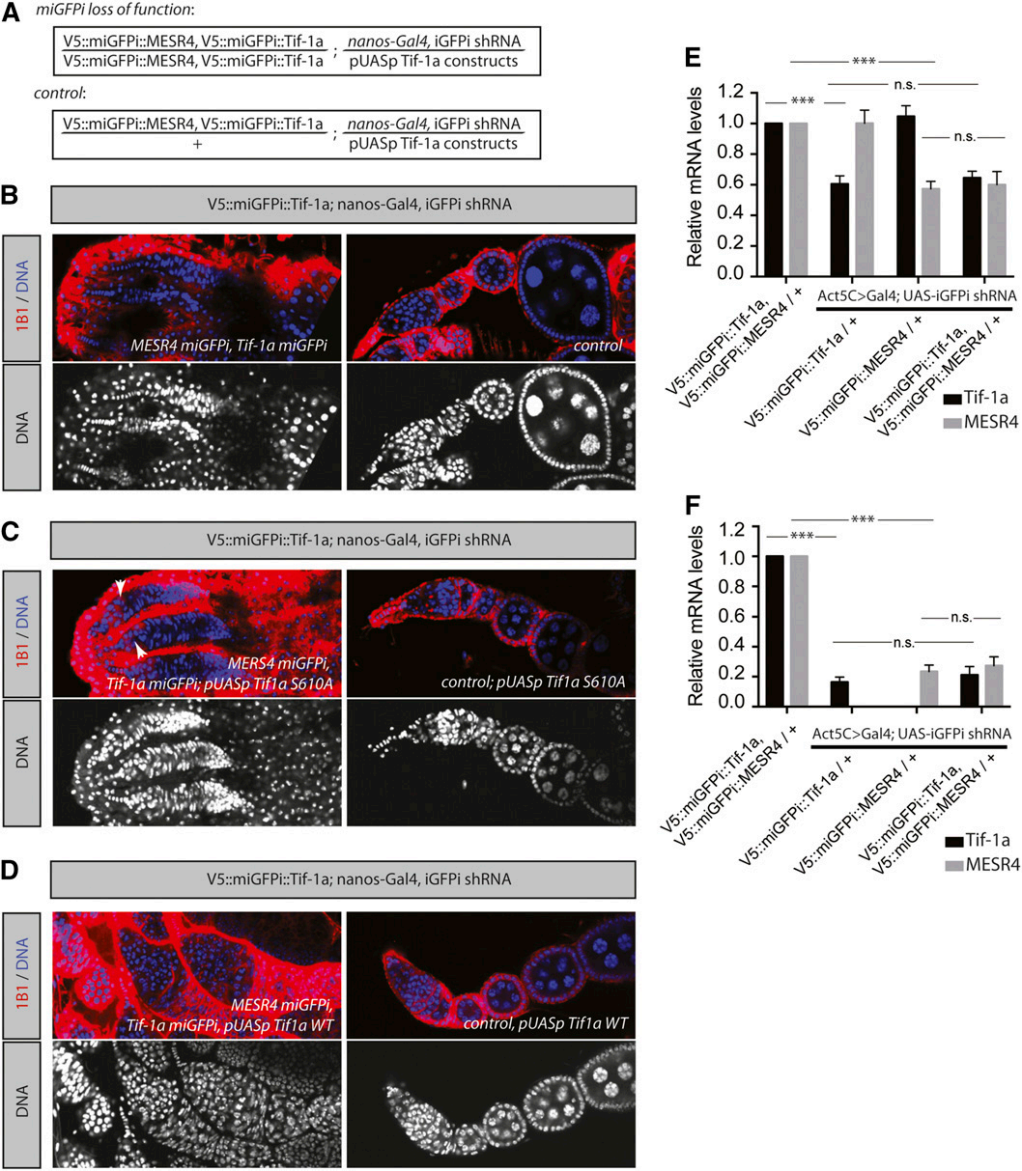
miGFPi allows for simultaneous knockdown of multiple genes with one validated shRNA line. (A) Outline of the miGFPi genotypes for the *Tif-1a*, *MESR4* double mutant experiments. (B–D) Combined loss of *Tif-1a* and *MESR4* function in the germline using miGFPi results in the *Tif-1a* phenotype and a loss of all germline cells. Expression of *Tif-1a* S610A in the *Tif-1a*, *MESR4* double miGFPi background results in the *Tif-1a* phenotype, whereas expression of *Tif-1a* WT results in the *MESR4* loss-of-function phenotype, demonstrating that miGFPi effectively depletes both genes. Red, 1B1; blue/gray, DNA. (E and F) qPCR validation of miGFPi mediated knockdown of *Tif-1a* and *MESR4* using gene-specific primers (E) and miGFPi allele-specific (F) primers. Error bars = SD. *** *P* < 0.001; n.s., not significant. Student’s *t*-test. GFP, green fluorescent protein; iGFPi, *in vivo* GFP interference; miGFPi, minimal *in vivo* GFP interference; qPCR, quantitative PCR; shRNA, short hairpin RNA; WT, wild type.

The presence of the same 21 nucleotide eGFP-derived sequence in multiple genes would, in theory, permit their efficient parallel knockdown with one single validated RNAi line. To test this idea, we constructed flies in which we inserted the same *V5*::miGFPi sequence in the *Tif-1a* and *MESR4* genes (Figure 5A). As expected, upon miGFPi-mediated double loss-of-function of *Tif-1a* and *MESR4*, we detected ovaries with no germline cells resembling the *Tif-1a* loss-of-function phenotype (Figure 5B). We next attempted to prove the depletion of *MESR4* in this background using a genetic approach. To this end, we crossed the *Tif-1a* WT construct into the combined loss-of-function background and detected the *MESR4* phenotype in these ovaries (Figure 5D). Conversely, the expression of the *Tif-1a* S610A construct caused an intermediate phenotype with the cooccurrence of empty ovarioles and ovarioles filled with poorly differentiated cells (Figure 5C). These experiments prove that both *Tif-1a* and *MESR4* function are lost in the parallel miGFPi approach. Consistently, quantitative real-time PCR with gene (Figure 5E) and miGFPi allele-specific primers (Figure 5F) confirmed the simultaneous knockdown of the two genes. Altogether, these data suggest that miGFPi can be used for the combined loss-of-function of multiple genes in parallel in a cell type-specific manner.

### miGFPi for the analysis of specific protein-isoforms

**Figure 6:**
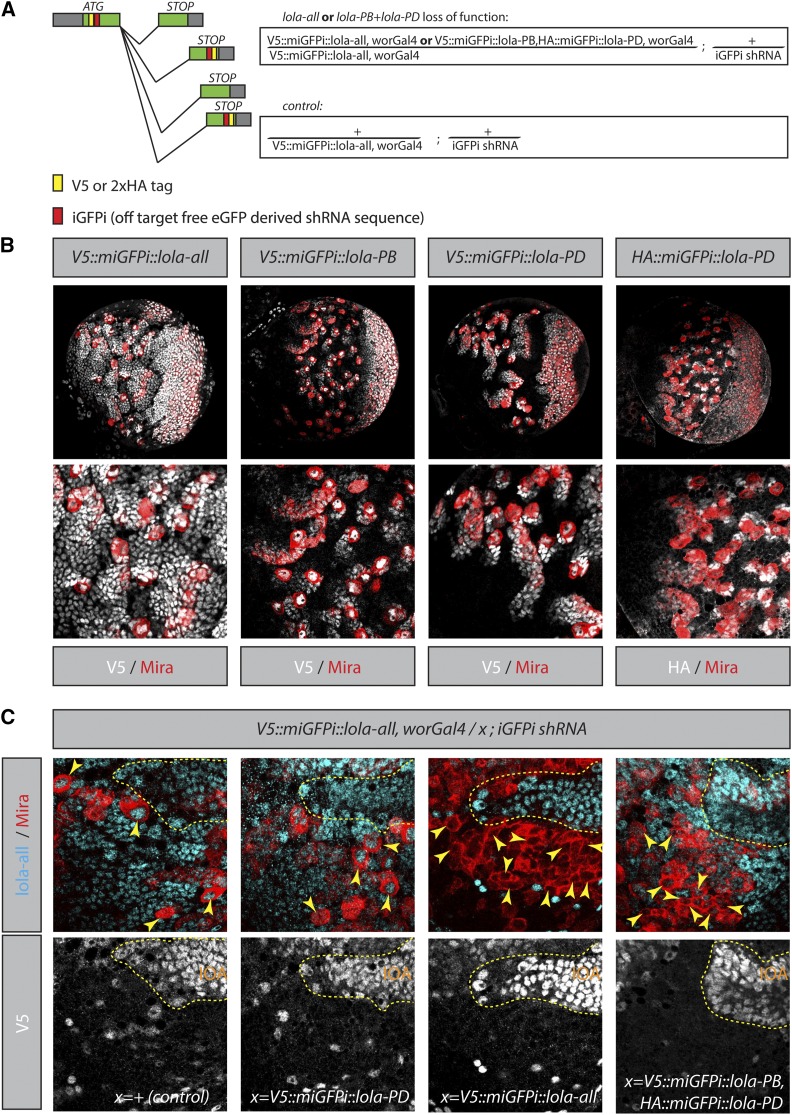
miGFPi allows for isoform-specific loss-of-function analysis. (A) miGFPi strategy for *lola*-all and simultaneous *lola*-PB and *lola*-PD loss-of-function. (B) lola is expressed in the larval brain. V5 staining of V5::miGFPi::*lola*-all, V5::miGFPi::*lola*-PB, and V5::miGFPi::*lola*-PD, and HA staining of V5::miGFPi::*lola*-PB and HA::miGFPi::*lola*-PD. (C) *lola* function is required for NSC differentiation. Depletion of all *lola*-isoforms or the simultaneous loss of the two most abundant NSC *lola*-isoforms, *lola*-PB and *lola*-PD (right), results in a NSC overproliferation phenotype at the posterior side of the larval central brain compared to the wild type (left). Individual loss-of-function of *lola*-PB and *lola*-PD does not induce NSC overproliferation, suggesting a complex interaction of these isoforms in NSCs. lola can be detected in the cells of the inner optic anlagen (IOA), where wor-Gal4 is not expressed. Red, Miranda; blue, lola-all; gray, V5. eGFP, enhanced GFP; GFP, green fluorescent protein; iGFPi, *in vivo* GFP interference; miGFPi, minimal *in vivo* GFP interference; NSC, neural stem cell; shRNA, short hairpin RNA.

Next, we asked whether it is feasible to functionally characterize individual isoforms of a gene by miGFPi. To this end, we focused on the BTB-Zn finger transcription factor *longitudinals lacking* (*lola*) in NSCs (Seeger *et al.* 1993; Giniger *et al.* 1994). The *lola* gene locus gives rise to 25 different splice isoforms that encode for 20 different proteins. All of them share a common N-terminal BTB domain but have different C-termini (Goeke *et al.* 2003; Ohsako *et al.* 2003) (Figure S3). In addition to its large number of isoforms, *lola* is a particularly exciting candidate to assess our miGFPi strategy, since ambiguity exists in the literature with respect to the function of *lola* in NSC cell fate specification in the central larval brain (Neumuller *et al.* 2011; Southall *et al.* 2014). Intriguingly, the expression of several independent shRNAs targeting the common N-terminal domain of *lola* result in phenotypes of variable strength (Figure S4B). To clarify *lola* function in central brain NSCs, we used the miGFPi approach. We integrated our miGFPi cassette in-frame into the common N-terminal domain of *lola*, thereby tagging all *lola* isoforms (Figure 6A). Additionally, we integrated the same sequence at the C-terminal domain of two *lola* isoforms, *lola-PB* and *lola-PD*. We decided to tag these isoforms as they are the most abundantly expressed isoforms in central brain NSCs (Table S2). We further generated V5 tagged *lola-PB*, and an HA tagged *lola-PD* isoform, with a common miGFPi sequence on the same chromosome for simultaneous knockdown (Figure 6B) and expression studies using V5 and HA-specific antibodies, respectively. All endogenously tagged flies are homozygous, viable, and fertile. Wor-Gal4-mediated miGFPi depletion of all *lola* isoforms (*lola-all*) results in overproliferation of NSCs at the expense of differentiating cells at the posterior side of the larval central brain (Figure 6C). The loss of all lola protein isoforms in central brain NSCs was verified by immunostaining with either an anti-V5 or an anti-lola antibody that recognizes the common N-terminal domain *(*Giniger *et al.* 1994*)*. In cells of the inner optic anlagen (IOA), where *wor*-Gal4 is not expressed, lola can be readily detected using anti-V5 or anti-lola antibodies, underlining the specificity of these reagents.

We next wanted to address if individual or combinatorial loss of the most highly expressed *lola* isoforms accounts for the observed phenotypes. Individual miGFPi of either *lola-PB* or *lola-PD* is not sufficient to promote overproliferation of NSCs (Figure 6C and Figure S4A). In contrast, the simultaneous loss of *lola-PB* and *lola-PD* resulted in a striking NSC overproliferation phenotype (Figure 6C). The low lola protein levels detected in Miranda-positive central brain NSCs demonstrate that other *lola*-isoforms are still expressed. Altogether, these data suggest that the simultaneous loss of the two most abundant NSC *lola*-isoforms is sufficient to induce an expansion of undifferentiated NSCs. The specific location of overproliferating NSCs at the posterior side of the brain suggests that *lola* is required in type II central brain NSCs to control self-renewal.

Altogether, these data suggest that the simultaneous expression of specific lola isoforms is required in type II NSCs to regulate stem cell differentiation. Thus, miGFPi is a powerful tool to rigorously study the function of individual protein isoforms and their combinatorial requirement in a cell type-specific manner.

## Discussion

In this study, we report a generalized strategy [minimal *in vivo* GFP interference (miGFPi)] for the characterization of gene function. We believe that this approach is applicable to all protein-coding genes and serves as a major improvement over other loss-of-function strategies, due to its versatility and standardized manner. We combine a CRISPR/Cas9-oligo based approach to integrate a small immunotag (V5 or HA) and a 21 nucleotide eGFP-derived sequence (encoded on one oligonucleotide) in-frame into the coding region of a gene or a specific isoform of a gene. While the immunotag allows for localization as well as biochemical studies, the 21 nucleotides serve as an RNAi effector sequence to conduct loss-of-function studies with highly validated RNAi reagents targeting this sequence in an off-target free manner.

The presented method has three key advantages over existing methods. Firstly, miGFPi, unlike its predecessor iGFPi, is not dependent on existing eGFP protein trap or MIMIC lines (Pastor-Pareja and Xu 2011; Caussinus *et al.* 2012; Neumuller *et al.* 2012). Because of the flexibility of the CRISPR/Cas9 system, the strategy can be used for all genes for which functional gRNAs can be identified. Therefore, intronless genes (or genes with small introns) which are *a priori* not accessible to iGFPi are amenable to miGFPi. In addition, the targeted insertion of the *miGFPi* tag into different isoforms of one gene allows for isoform-specific loss-of-function in a single or combined manner. Secondly, miGFPi in the herein presented form represents an “all-in-one” strategy, such that it covers many potential experimental procedures for the functional characterization of gene function with one single step of reagent generation due to the availability of excellent, specific, and standardized reagents. Finally, miGFPi is a highly time efficient method that allows for phenotypic characterization within weeks in a medium throughput manner.

Importantly, miGFPi allows for very flexible genetics and the construction of complex genetic experiments. We exemplify this by the combined loss-of-function of *Tif-1a* and *MESR4* with one RNAi construct, and the parallel introduction of *Tif-1a* rescue or point mutant constructs. Although not explicitly tested in this study, we envisage that the parallel loss of a larger set of genes or protein isoforms might be possible using miGFPi. Therefore, miGFPi serves as a fast strategy for the characterization of gene function in *Drosophila* and other species for which CRISPR/Cas9 mediated tagging is possible, and highly specific RNAi reagents against the tag (that do not induce phenotypes by themselves) can be established.

Moreover, miGFPi itself is very versatile and can be adapted to different needs; besides the V5 tag, alternative immuno tags can be used. For example, we introduced a HA tag in one of the *lola* isoforms. In addition, different RNAi effector sequences, optimal for cell types other than GSCs and NSCs, can be readily identified. It might even be possible to direct off-target free RNAi directly against the immunotag-encoding nucleotide sequence. This would further decrease the size of the exogenous sequence introduced at the genomic locus of interest.

In this study, we further describe functions for three genes in different stem cell compartments: We show unequivocally that the Pol I-specific transcription factor *Tif-1a* is required for the maintenance of GSCs. The Grummt lab has demonstrated that human Tif-1a is a phosphoprotein that integrates input from signaling cascades and translates this input into a transcriptional response from the rDNA gene cluster (Zhao *et al.* 2003). Serine 649 is phosphorylated by ERK in situations of increased cell growth. Starving cells conversely show low levels of S649 phosphorylation (Zhao *et al.* 2003). This phosphorylation site is functionally required for Tif-1a function as a S649A mutation abrogates Pol I transcription (Zhao *et al.* 2003). Similar to its human homolog, we show in this study that *Drosophila* *Tif-1a* is a phosphoprotein and that Serine 610 is phosphorylated in *Drosophila* S2 cells. Using miGFPi, we were able to show that this phosphorylation site is functionally required for *Tif-1a* function in GSCs.

In addition, we characterize the function of *MESR4* in this study. Consistent with the presence of a plant homeodomain and several zinc fingers, *MESR4* localizes predominantly to the nucleus. In the female *Drosophila* GSC lineage, MESR4 is predominantly expressed in differentiating cells. *MESR4* is expressed at low levels in GSCs and early cystocytes but strongly induced in 16-cell cystocytes, suggesting a role in later steps of germline development. Indeed, loss of *MESR4* function is associated with differentiation defects and the absence of mature oocytes. Besides an expansion of cystocytes in *MESR4* miGFPi ovarioles, we detected a defect in Orb localization, demonstrating that mature oocytes are absent in *MESR4* germline-depleted ovaries. These data suggest that *MESR4* controls multiple processes in germline cell development. It will be interesting to determine the relevant target genes of *MESR4* that regulate these cell fate programs.

Lastly, by using miGFPi, we clarify the function of *lola* in NSCs of the central brain and show that *lola* function is required for type II NSC differentiation. A previous study was unable to detect a requirement of *lola* in central brain NSCs by using mosaic clonal analysis (Southall *et al.* 2014). The overproliferation phenotype might have been missed due to the spatiotemporal variability of the method. Using miGFPi, we can unambiguously demonstrate that specific *lola* isoforms are expressed and functionally required for central brain NSC self-renewal. We tagged the common N-terminal domain of *lola* and two of the 25 different isoforms and showed that the simultaneous expression of the most highly expressed isoforms, *lola-PB* and *lola-PD*, is required for proper NSC differentiation. Interestingly, individual loss of these isoforms does not induce NSC overproliferation, suggesting a complex interplay of *lola* isoforms in NSCs.

Altogether, we describe a novel strategy, miGFPi, which we believe will greatly facilitate the characterization of gene function in a stringent and exceptionally time effective manner. miGFPi is a highly flexible method and we envisage broad applications of this strategy beyond its use in *Drosophila*.

## Supplementary Material

Supplemental Material
