# Supplementary material for: A Combination of CRISPR/Cas9 and Standardized RNAi as a Versatile Platform for the Characterization of Gene Function
Source: G3 (Bethesda). 2016 Jun 7;6(8):2467–78. doi: 10.1534/g3.116.028571 (PMC4978900; doi:10.1534/g3.116.028571)
Supplement: Supplemental Material [file supp_6_8_2467__index.html]

A Combination of CRISPR/Cas9 and Standardized RNAi as a Versatile Platform for the Characterization of Gene Function — A Combination of CRISPR/Cas9 and Standardized RNAi as a Versatile Platform for the Characterization of Gene Function — Supplemental Material 

# A Combination of CRISPR/Cas9 and Standardized RNAi as a Versatile Platform for the Characterization of Gene Function

## Supplemental Material for Wissel, *et al*, 2016

**Files in this Data Supplement:**

- File S1 - This file contains the legends for all Supplemental Figures. (.pdf, 4.7 MB)
- Figure S1 - Drosophila Tif-1a is a phosphoprotein. (.pdf, 898 KB)
- Figure S2 - MESR4 RNAi phenotypes resemble MESR4 miGFPi. (.pdf, 226 KB)
- Figure S3 - *lola* isoform composition. (.pdf, 373 KB)
- Figure S4 - lola phenotypic analysis. (.pdf, 2.1 MB)
- Table S1 - Sequences of oligonucleotides used in this study. (.xlsx, 15 KB)
- Table S2 - Expression of *lola* isoforms in NSCs and neurons. (.xlsx, 10 KB)
