# Supplementary material for: A Combination of CRISPR/Cas9 and Standardized RNAi as a Versatile Platform for the Characterization of Gene Function
Source: G3 (Bethesda). 2016 Jun 7;6(8):2467–78. doi: 10.1534/g3.116.028571 (PMC4978900; doi:10.1534/g3.116.028571)
Supplement: Supplemental Material [file supp_g3.116.028571_FileS1.pdf]

A

MS/MS-spectrum of the C-terminal phosphopeptide Tif-1a

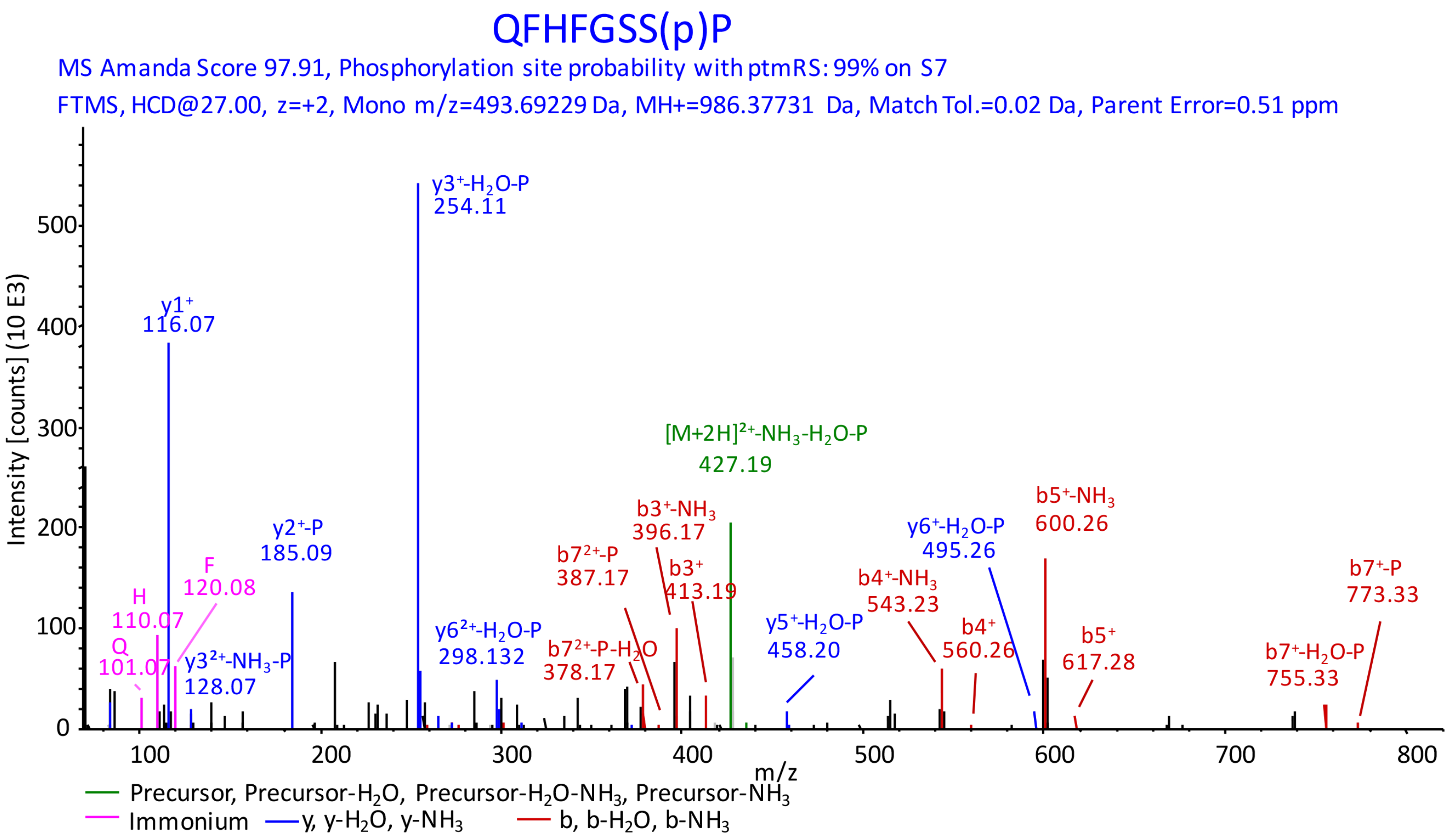


B

MS/MS-spectrum of the synthetic **phosphopeptide**

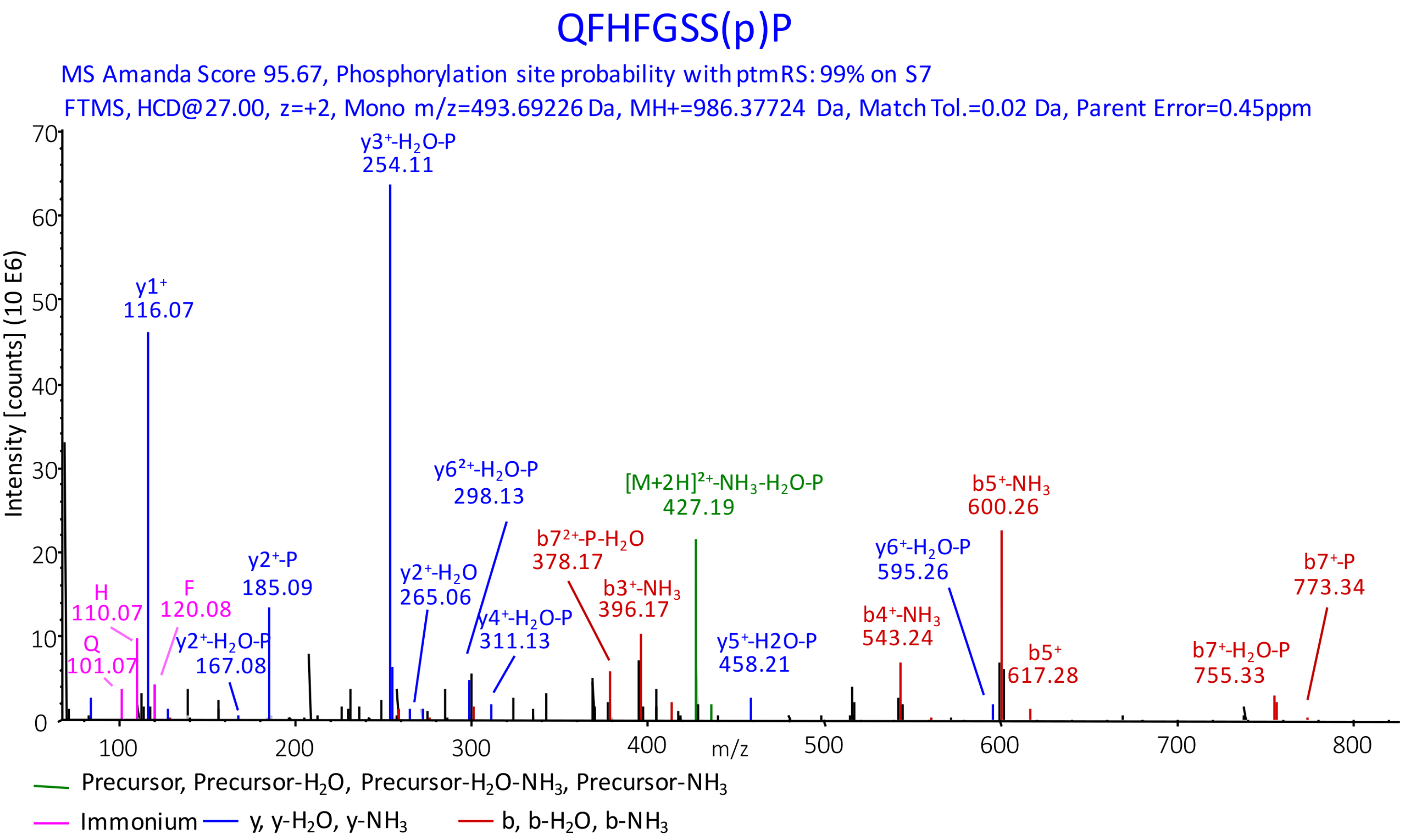

**Supplemental Figure 1. Drosophila Tif-1a is a phosphoprotein**

Spectra of c-terminal Tif-1a peptides demonstrating phosphorylation of S610A. Phosphorylation status was determined comparing endogenous Tif-1a to synthetic peptides of the same sequence that are phosphorylated or not phosphorylated.

A

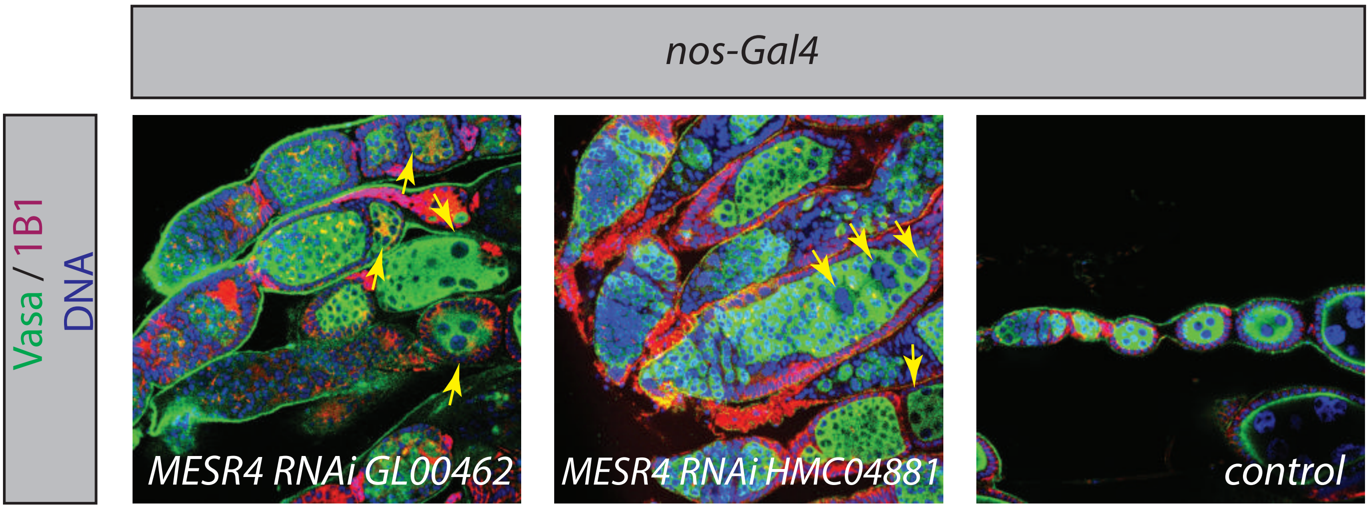


**Supplemental Figure 2. MESR4 RNAi phenotypes resemble MESR4 miGFPi**

A) Knockdown of MESR4 with two independent shRNA constructs results in an expansion of undifferentiated 1B1 positive cells in the germarium and pseudo egg chambers that are filled with 1B1 positive or polyploidy cells (yellow arrows). Compared to control flies, mature eggs are not detectable upon MESR4 knockdown. Red: 1B1, Blue: DNA, Green: Vasa.

longitudinals lacking (*lola*):

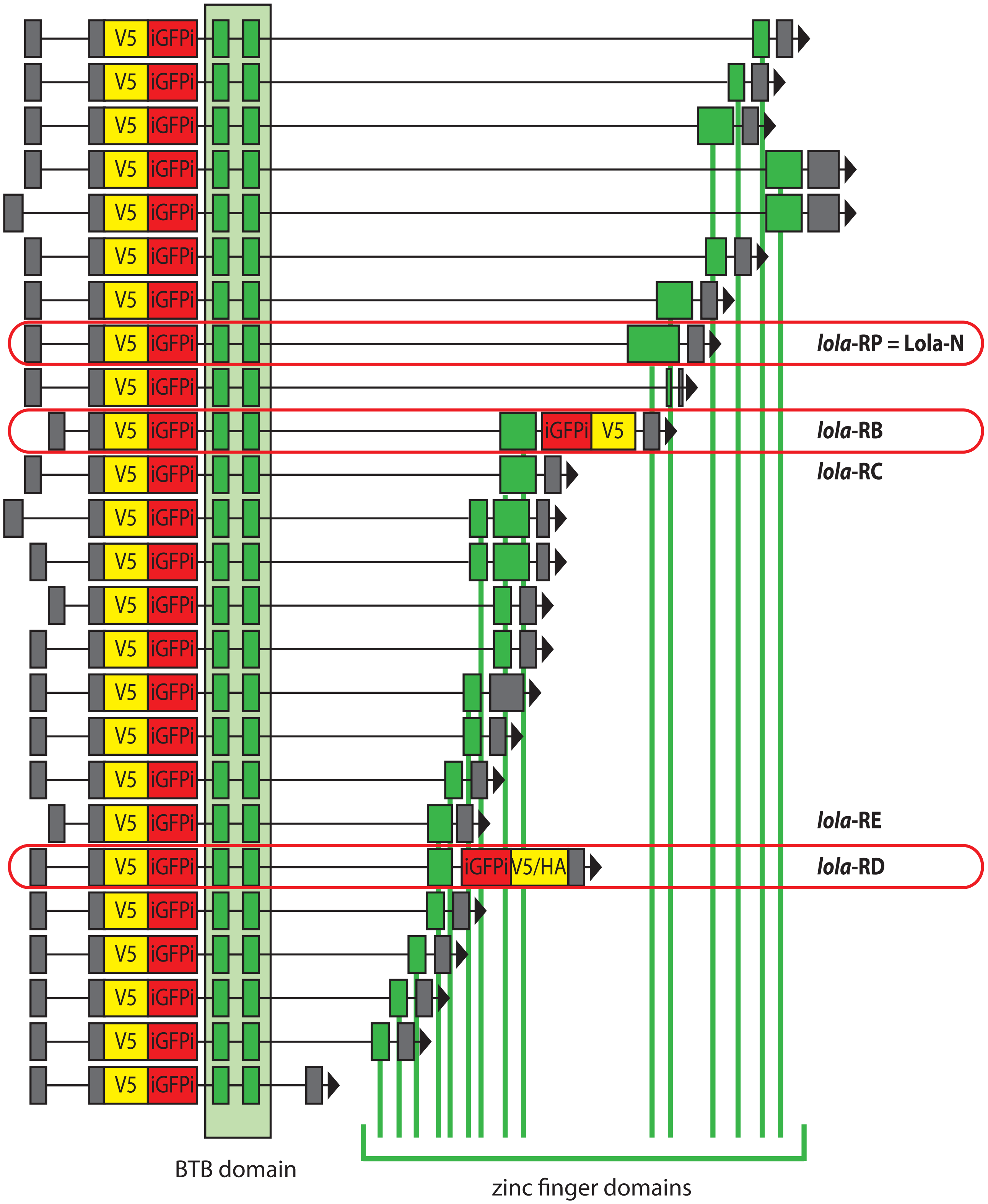


| isoform | FPKM Neuroblast | FPKM Neuron |
|---|---|---|
| *lola*-RB/RC | 66.8 | 46.5 |
| *lola*-RD/RE | 163.6 | 74.2 |
| *lola*-RP = lola-N | 25.9 | 227 |

Data from Berger et al. 2012

**Supplemental Figure 3. *lola* isoform composition**

For *miGFPi* of all *lola* isoforms we inserted a V5 tag followed by the *iGFPi* sequence at the n-terminal region of the gene. For isoform specific loss-of-function we inserted the *iGFPi*-V5 sequence into the open reading frame at the c-terminus. The table shows the expression levels (FPKM values) of *lola* isoforms *lola-RB*, *lola-RD* and *lola-N* in FACS sorted central brain neural stem cells (neuroblasts) and neurons.

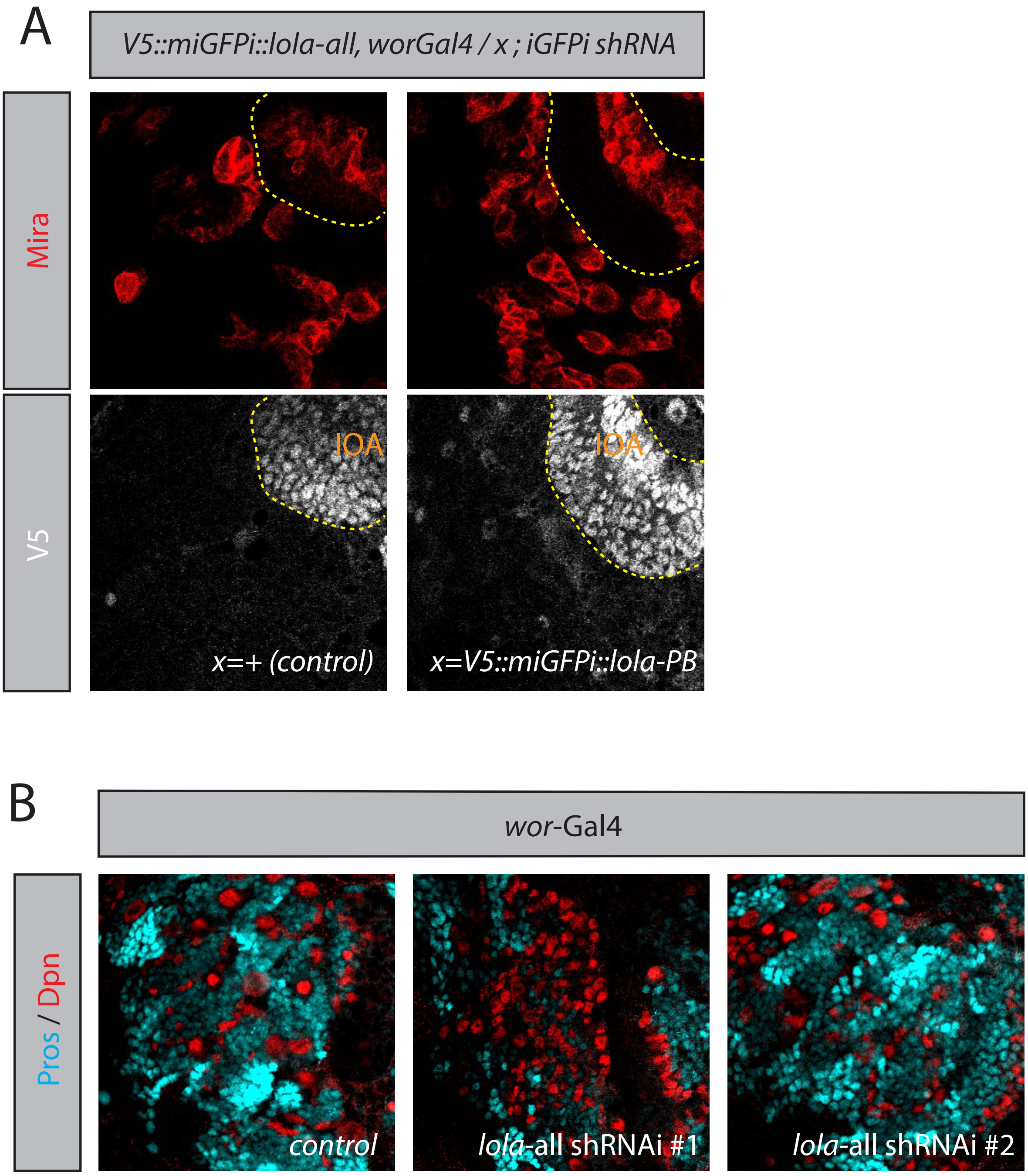


**Supplemental Figure 4. lola phenotypic analysis**

A) Individual miGFPi of lola-PB does not result in an overproliferation phenotype. Red: Miranda, Grey: V5

B) Examples of varying phenotypic strength resulting from lola-all knock-down with two independent shRNA constructs targeting the n-terminal part of lola that is common to all isoforms. Red: NSCs (Deadpan), cyan: neurons (Prospero).
