# Supplementary material for: A Combination of CRISPR/Cas9 and Standardized RNAi as a Versatile Platform for the Characterization of Gene Function
Source: G3 (Bethesda). 2016 Jun 7;6(8):2467–78. doi: 10.1534/g3.116.028571 (PMC4978900; doi:10.1534/g3.116.028571)
Supplement: Supplemental Material [file supp_g3.116.028571_FigureS1.pdf]

A

MS/MS-spectrum of the C-terminal phosphopeptide Tif-1a

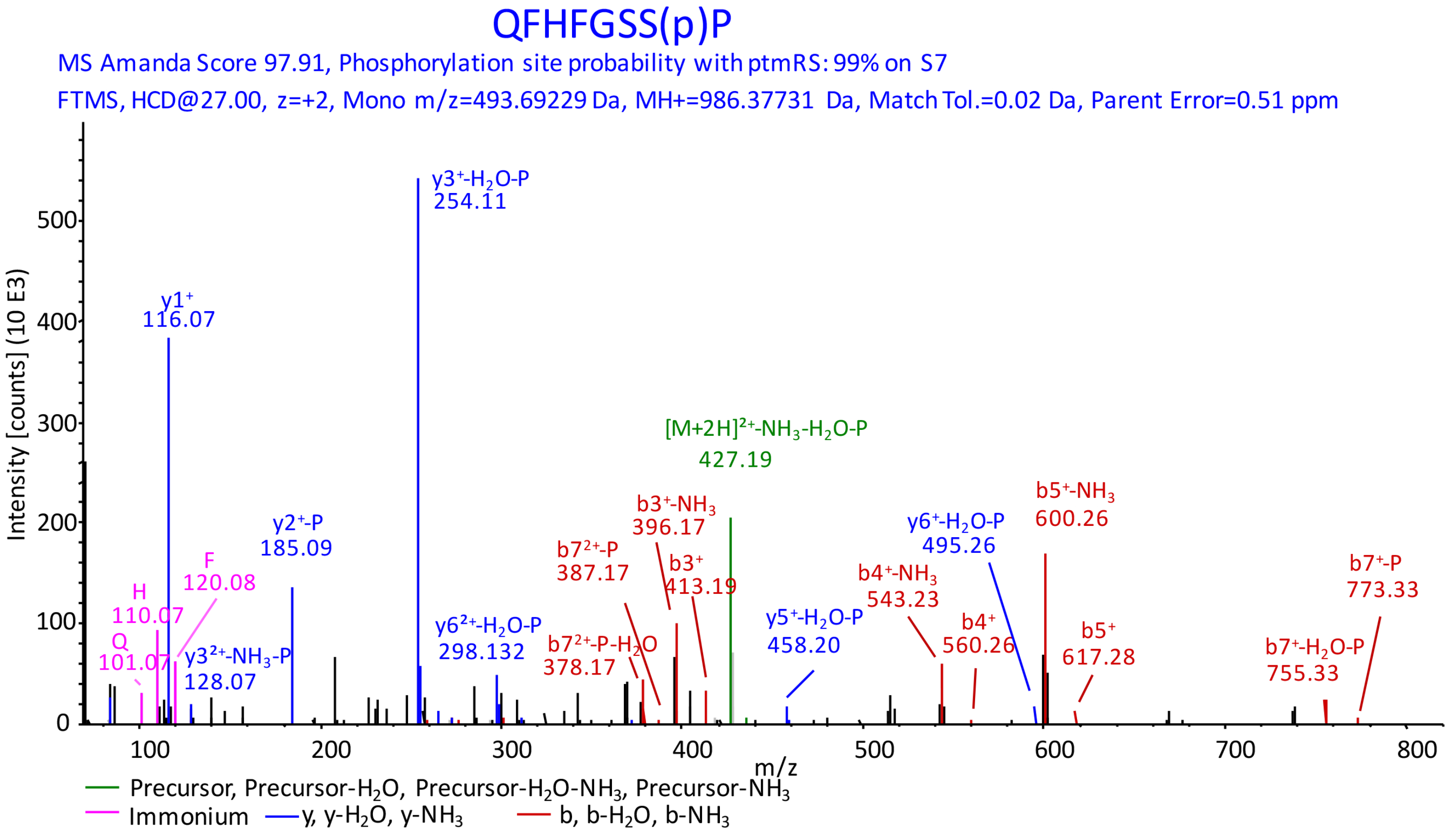

B

MS/MS-spectrum of the synthetic **phosphopeptide**

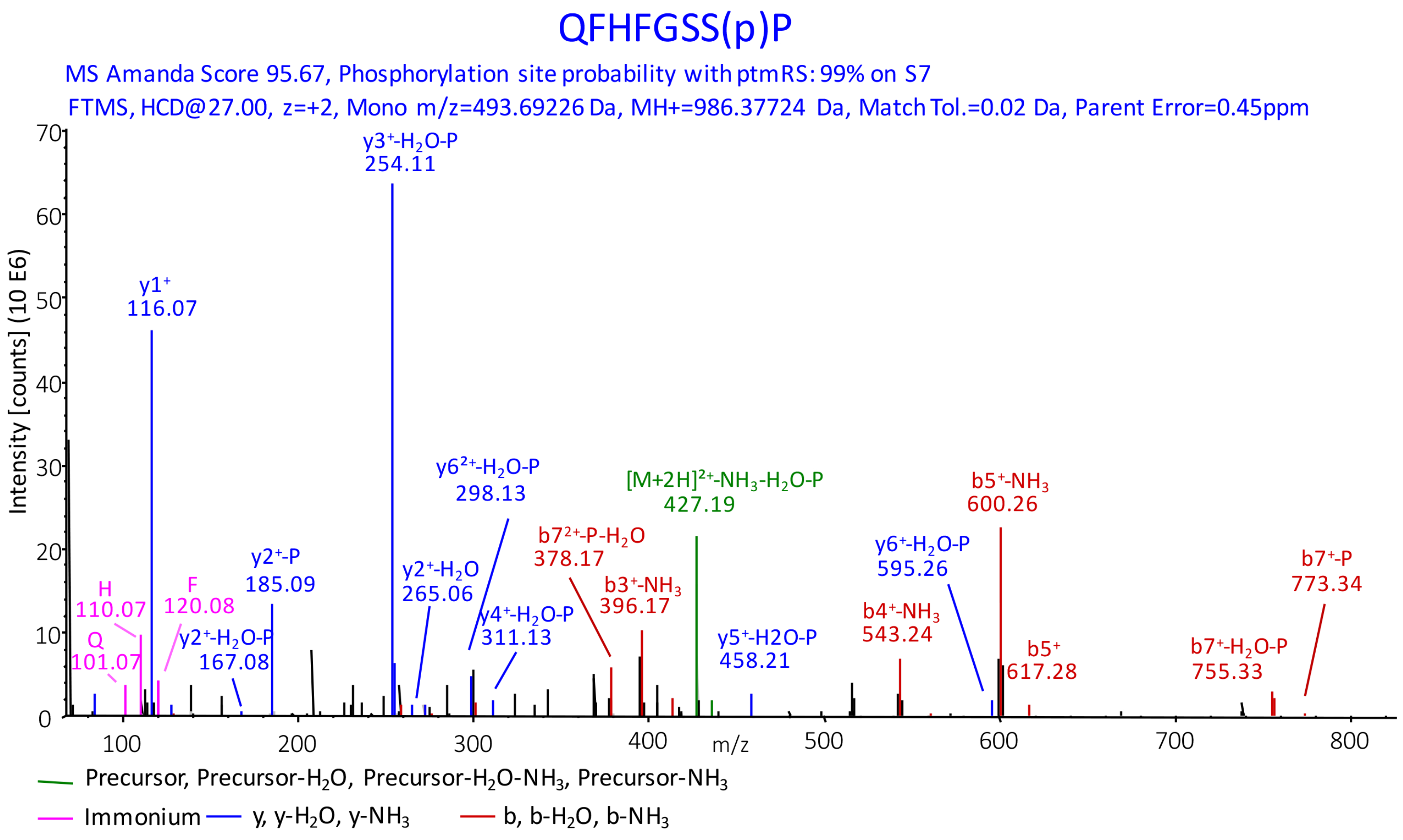

Wissel et al. Figure S1
