# Supplementary figures and images for: A Combination of CRISPR/Cas9 and Standardized RNAi as a Versatile Platform for the Characterization of Gene Function
Source: G3 (Bethesda). 2016 Jun 7;6(8):2467–78. doi: 10.1534/g3.116.028571 (PMC4978900; doi:10.1534/g3.116.028571)

A

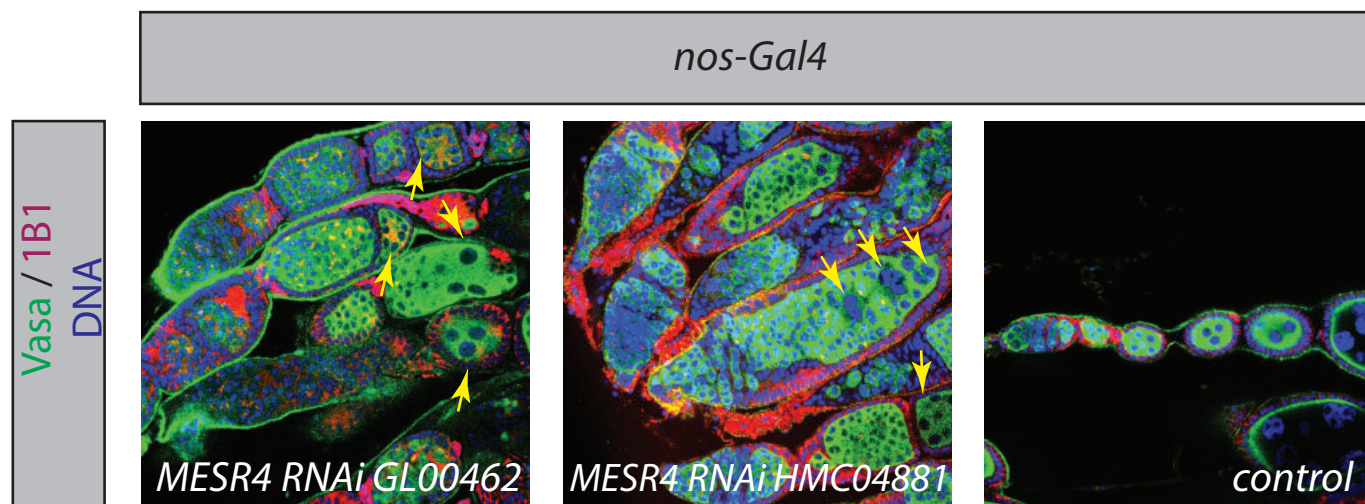

Wissel et al. Figure S2

Supplement: Supplemental Material [file supp_g3.116.028571_FigureS2.pdf]

A

*V5::miGFPi::lola-all, worGal4 / x ; iGFPi shRNA*

Mira

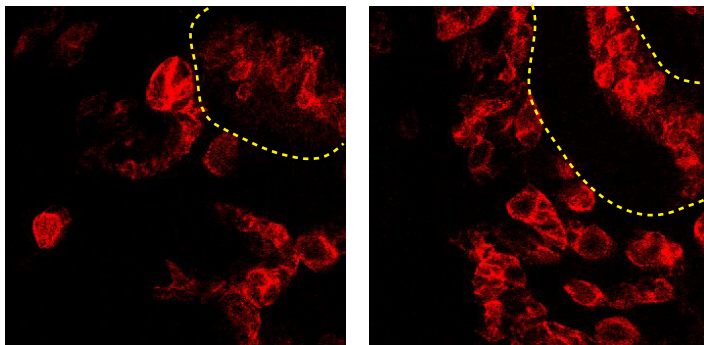

V5

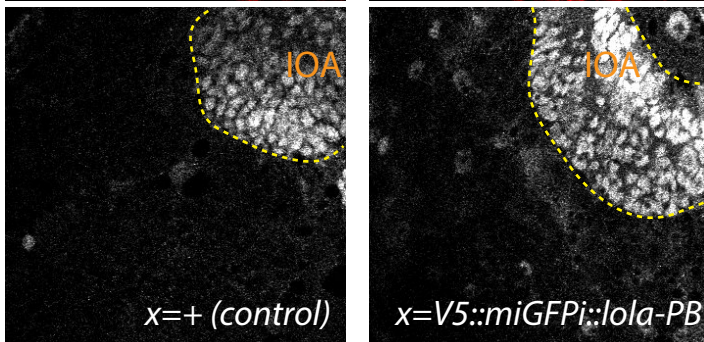

B

*wor-Gal4*

Pros / Dpn

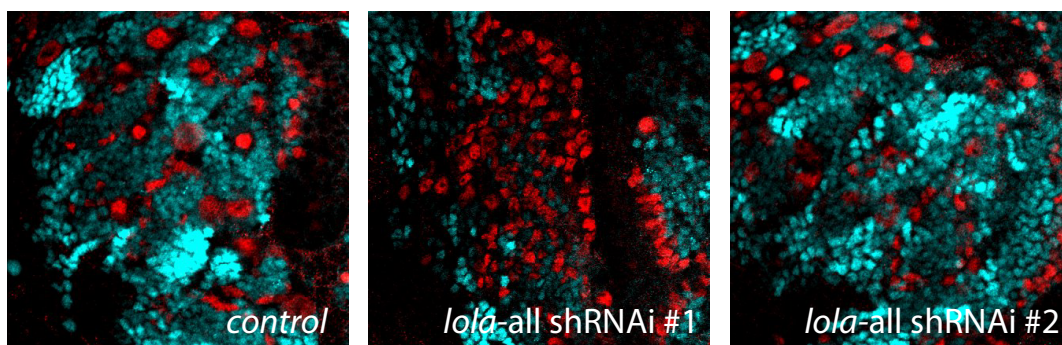

Wissel et al. Figure S4

Supplement: Supplemental Material [file supp_g3.116.028571_FigureS4.pdf]
