# Supplementary material for: A Combination of CRISPR/Cas9 and Standardized RNAi as a Versatile Platform for the Characterization of Gene Function
Source: G3 (Bethesda). 2016 Jun 7;6(8):2467–78. doi: 10.1534/g3.116.028571 (PMC4978900; doi:10.1534/g3.116.028571)
Supplement: Supplemental Material [file supp_g3.116.028571_FigureS3.pdf]

longitudinals lacking (*lola*):

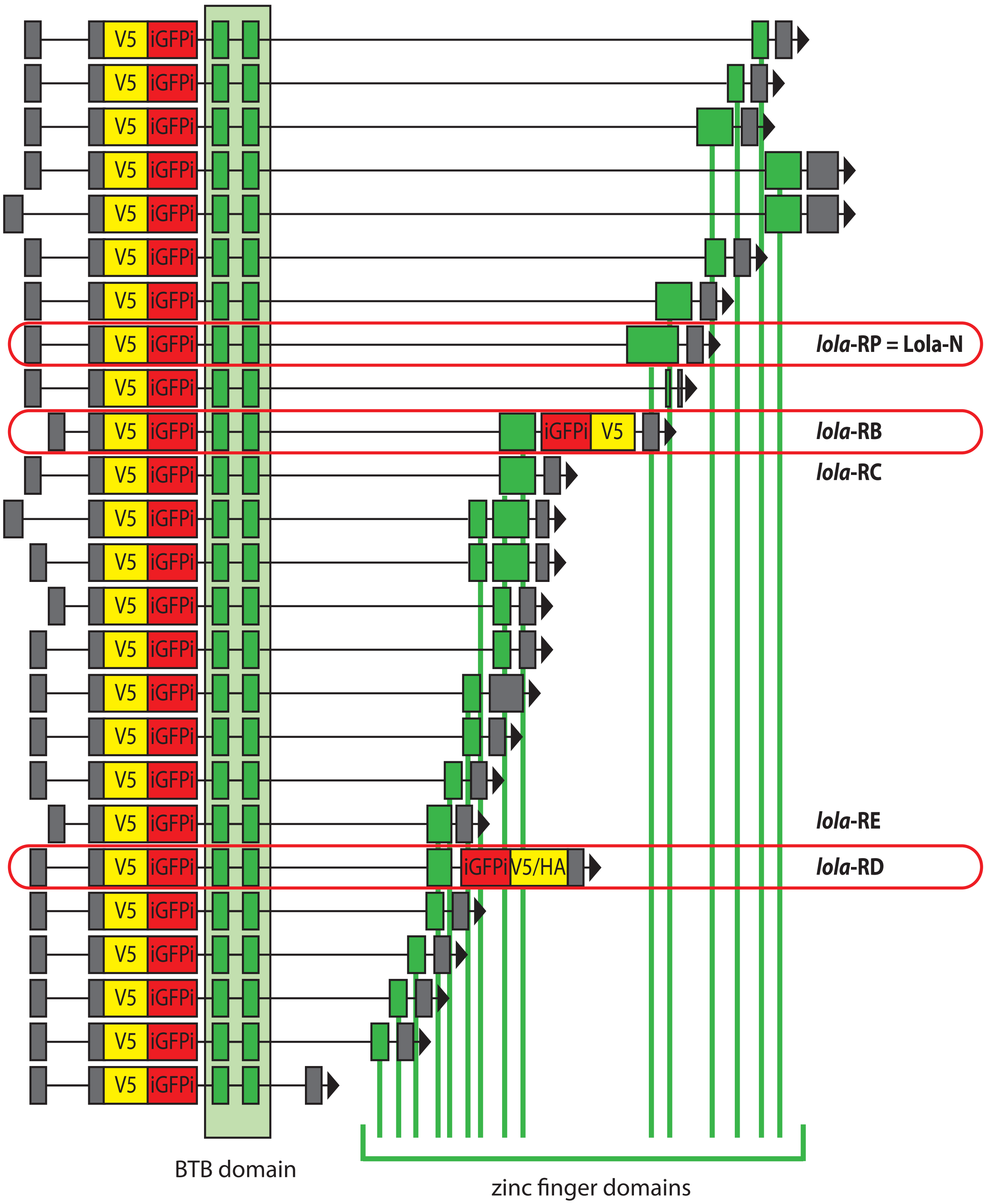

isoform	FPKM Neuroblast	FPKM Neuron
lola-RB/RC	66.8	46.5
lola-RD/RE	163.6	74.2
lola-RP = lola-N	25.9	227

Data from Berger et al. 2012

Wissel et al. Figure S3
